# Biodiversity, drug discovery, and the future of global health: Introducing the biodiversity to biomedicine consortium, a call to action

**DOI:** 10.7189/jogh.07.020304

**Published:** 2017-12

**Authors:** Vidushi Neergheen-Bhujun, Almas Taj Awan, Yusuf Baran, Nils Bunnefeld, Kit Chan, Thomas Edison dela Cruz, Dilfuza Egamberdieva, Simon Elsässer, Mari–Vaughn V. Johnson, Shoji Komai, Andrey L. Konevega, John H. Malone, Paul Mason, Rothsophal Nguon, Ross Piper, Uttam Babu Shrestha, Milica Pešić, Alexander Kagansky

**Affiliations:** 1 University of Mauritius, Réduit, Moka, Mauritius; 2 Federal University of Săo Carlos, Săo Carlos, Brazil; 3 Izmir Institute of Technology and Abdullah Gul University, Izmir, Turkey; 4 Stirling Conservation Science, Stirling University, Striling, UK; 5 Centre for Global Health Research, The Usher Institute for Population Health Sciences and Informatics, The University of Edinburgh, Scotland, UK; 6 University of Santo Tomas, Manila, Philippines; 7 National University of Uzbekistan, Tashkent, Uzbekistant; 8 Karolinska Institute, Stockholm, Sweden; 9 United States Department of Agriculture, Washington, D.C., USA; 10 Nara Institute of Science and Technology, Ikoma, Japan; 11 Petersburg Nuclear Physics Institute, Petersburg, Russia; 12 University of Connecticut, Farmington, Connecticut, USA; 13 School of Social Sciences, Monash University, Melbourne, Australia; 14 Pańńāsāstra University, Phnom Penh, Cambodia; 15 University of Leeds, Leeds, UK; 16 University of Southern Queensland, Toowoomba, Queensland, Australia; 17 University of Belgrade, Belgrade, Serbia; 18 University of Edinburgh, Edinburgh, UK and Far Eastern Federal University, Russia

Looking to nature for medicine is nothing new – we have been doing it for tens of thousands of years and although modern pharmaceutical science has come a long way from those ancient roots, nature is and will always be an important source of useful compounds and inspiration. Dismissing nature in this regard is a huge mistake as evolution is the greatest problem solver and the myriad compounds produced by the immense variety of species we share the planet with have been honed by three billion years of trial and error. However, with every bit of habitat that disappears under the plough or concrete we impoverish nature and deprive ourselves of potential medicines.

The preservation of biodiversity is perhaps the single most important building block for achieving the 17 Sustainable Development Goals set by the United Nations. For many of these goals, the importance of preserving biodiversity is obvious, e.g.SDG-2 (Zero Hunger), SDG-13 (Climate Action), SDG-14 (Life below Water), and SDG-15 (Life on Land). This argument holds true for the other global goals including, but not limited to, SDG-3 (Good Health and Well-being), SDG-10 (Reducing Inequalities), and SDG-12 (Responsible Consumption and Production). Preserving biodiversity in many landscapes and natural habitats free for people to enjoy and access both locally and worldwide, rather than only in museum collections and zoos is critical for understanding life, the workings of the biosphere and for developing methods to sustain the quality and longevity of human life. Of comparable importance, access to biodiversity as a living, evolving aspect of our planet has the potential to increase the public’s appreciation for these systems and processes.

Preserving biodiversity is in our self-interest. Nowhere does this ring truer than in drug discovery. The preservation of biodiversity provides a vital link to critically expand the molecular diversity necessary for successful drug discovery efforts in the future. Drug discovery from wild species has always been, and will continue to be one of the most critical for most if not all aspects of health care, disease prevention, and wellness [1]. In addition, chemical reagents, whether from natural or synthetic sources, are non-renewable, and using these reagents depletes future resources. Resources and knowledge (both traditional and modern scientific) about the ecology, taxonomy and usage of medicinally important organisms are too precious to squander. Consequently, all drug discovery programs, synthetic or natural, need to build the concept of sustainability into their research models.

Biodiversity therefore becomes critical to future drug discovery yet, there is alarming loss of biodiversity. Modern extinction rates are about 100 to 1000 times greater than extinction rates calculated over past eras [2]. Though new species are regularly discovered, known species go extinct at a rate 1000 times higher than the discovery of new species [3]. This ongoing loss of biodiversity is altering ecosystem functions and the ability to provide goods and services for human health and well-being. In the case of drug discovery, according to some estimates, our planet is losing at least one important drug every two years [4]. Further, the irreversible loss of traditional knowledge on the medicinal use of plants and animals and the loss of molecular diversity is concomitant with the extinction of microbes, plants, fungi, and animals. The complementary losses threaten biomedical research, and in turn, the survival of humans.

The sustainable development of natural products will not be possible without taking biodiversity conservation into consideration. While plants are commonly used for medicinal purposes, new possibilities are emerging from organisms that are incredibly diverse biologically and chemically, but relatively understudied, such as arthropods and fungi, particularly in many countries deemed as ‘biodiversity hotspots’ [5]. We can be certain that we share the planet with an enormous variety of species. A very recent estimate of 1-6 billion species is certainly realistic when we take into account parasites, parasitoids and endosymbionts [6]. Knowing exactly where to look among all of this life, especially the hyper-diverse tax, and obtaining sufficient quantities of starting material have been an issue, historically, but new approaches and technology will surmount these stumbling blocks. Overall, the limitations of combinatorial chemistry and high-throughput screening, together with the promise of phenotype-based screening, transcriptomics and synthetic biology suggest the arrival of a new era of drugs derived from natural extracts [7–10]. We believe that collecting, curating, and disseminating knowledge on biodiversity as it relates to the treatment of human diseases will promote the conservation of bio- and molecular diversity and, simultaneously, create the international cooperation needed to safeguard well-being for all communities.

Photo: Illustration created by Milica Pešić using CC0 Creative Commons images (no attribution required).

Sound ethical oversight and responsible policy implementation needs to accompany exploration of medicinal species, whether bacteria, fungi, plants or animals, for drug discovery. In developing and low-income countries, there is a significant amount of biodiversity that is understudied and available for exploration. These areas are likely to be under disproportionate pressure as medical research turns to biodiverse areas for new drugs. Conversely, developed nations have lost biodiversity while achieving economic progress [11]. Losing novel molecules at the expense of economic progress undermines solutions for human health and economic revenue for the communities where biodiversity should be protected most.

Future efforts to explore biodiversity for drug discovery must consider the interests of indigenous people, respect for their knowledge, and those living in developing, low-income countries. In developing countries, plants are a primary source of health care. When large pharmaceutical companies obtain medicinal plants or purchase lands that support their native habitat in order to make new drugs, these drugs and the plants themselves can become unavailable or unaffordable to the local people who will have no means to buy the products that are developed from these sources. Local plants, which contain mixtures of phytochemicals used as herbal medicines, are far less expensive, and often more available to economically challenged communities than are compounds that are isolated, purified, standardized, and subjected to clinical trials. In addition, local communities may suffer because they may be displaced from culturally important traditional lands. Further, traditional knowledge of species properties and preparations may be lost as medical research forces “western” values and approaches to medicine on diverse local populations.

At both the local and global scales, we should not satisfy our own needs at the expense of future generations, or indeed of other species on Earth. Ensuring the health and safety of other species is in our interests as a healthy planet for humans relies on a rich variety of species. Furthermore, ecosystem governance is also required if significant problems in biomedicine are to be solved through the analysis of global biodiversity.

To safeguard indigenous communities, protect biodiversity, and sustainably pursue drug discovery for the benefit of everyone, it is of paramount importance to strengthen the international implementation of the following practices:

- Investigate and standardise natural products: therapeutic potential, chemistry, ecology, availability and potential to cultivate, traditional use, *in situ* and *ex situ* conservation, sustainable trade, and impacts by climate change, with special focus on indigenous medicinal species with potential therapeutic properties.
- Implement ethical and governance models to engage with diverse indigenous communities to collect existing knowledge on species of interest, and create online databases for easy access, dissemination, and equitable distribution of benefits.
- Promote open inter-disciplinary domestic and international dialogue and information sharing among academics, physicians, patients, policy-makers, commercial bodies, and local and indigenous community stakeholders in the areas of medicine, health and wellbeing, with special focus on understanding different cultural norms and language needed to describe traditional medicine.
- Establish best practices for sustainable natural product collection, production, storage, and preparation–with special attention to safeguard traditional family preparations and assurance that value is returned to local communities, and standardise high capacity bio-molecular and cell-based assays in testing these natural products.
- Raise awareness of the long-term economic benefits of protecting biodiversity over the short-term benefits of habitat destruction and unsustainable resource extraction.
- Promote best practices in sustainable commercialisation of natural products that consider the balance of ecosystems and population needs and the implementation of a fair and equitable share of benefits among current and future stakeholders.

An urgent international and interdisciplinary initiative is required to promote partnerships among diverse stakeholders high, middle, and low-income countries to protect existing knowledge and biodiversity and to support scientists in evaluating natural product-based therapeutic agents in a standardised and sustainable manner. Formation of multilateral, multidisciplinary research consortia that are global in scope is the pressing need to achieve these goals. Further, public-private partnerships will likely play key roles in addressing this challenge.

New funding models by international and government agencies, pharmaceutical companies, academic institutions, non-governmental organisations, scientific societies, and private foundations/donations are needed to promote work on:

- Collecting, curating, and disseminating information locally, regionally, and globally, from various disciplines regarding historical, current, and potential use of the remaining species for control and prevention and treatment of human diseases in order to promote research, protection, conservation, and international cooperation.
- Connect governmental, research and medical organisations focusing on collecting and testing natural products, developing biological and biomedical assays, health/nutrition regiments, ecological protocols, policy development, and such, to create a common understanding, standard protocols, and best practices in natural treatment and drug development in the interest of the survival of humans and other species.

**Figure 1:**
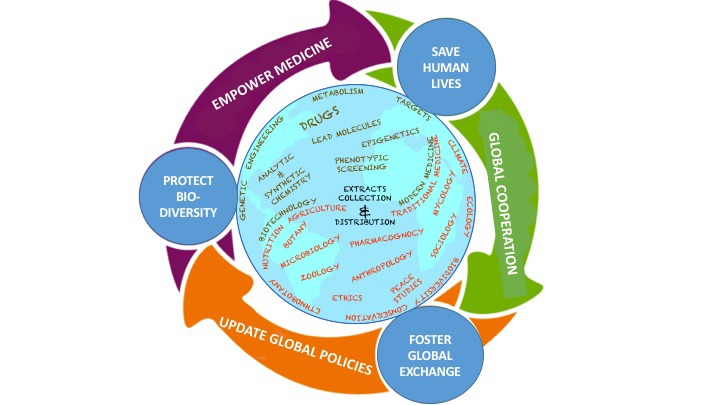
Schematic representation of the global and interdisciplinary nature of our proposal. Names of the selected research subjects to be included in our consortium are indicated over the globe/map of the world in the shape of 'Tao', with disciplines being distributed according to their relation to each other, in the context of our initiative, to form two large interconnected groups 'Global South' and 'Global North', characteristic by overall tendency to 'biodiversity and traditional knowledge' and 'technology and modern knowledge', respectively. The metaphorical relation to the ancient and modern Chinese symbol of 'Tao' is demonstrating the holistic or 'non-dualistic' approach to the solution to biodiversity and biomedicine problems, requiring acknowledgement and cooperation of bigger scientific community in order to promote mutually beneficial co-existence of humankind with other species.

In order to achieve the above goals, we have established a consortium of early-career scientists representing a wide range of disciplines and countries (**Figure 1**). The Bio2Bio (Biodiversity-to-Biomedicine) consortium will 1) promote exchange of traditional and modern knowledge across disciplines and borders, 2) build a unified framework for sharing resources and data while conforming to international treaties and local regulations, and 3) create an interdisciplinary knowledge hub to communicate research and empower the public, physicians, patients, and policymakers to create a unified approach to selecting, protecting, and undertaking research of the remaining wild species on our planet.

In conclusion, it is crucial that governments, global organizations, and local stakeholders come together to agree on preservation of remaining hotspots of biodiversity through development of partnerships. Activities should include the collation and generation of knowledge about the regions and their species’ potentials and provide education and collaborative outreach to local governments, decision makers, and stakeholders.

This largely academic group will also seek to engage individuals and organisations outside of academia via our extended professional networks to help meet the objectives of the consortium. In addition, we invite new members, contributors, and funding opportunities. If you would like to learn more, please contact us at info@bio2bio.net. Together, we can create a new paradigm in protecting biodiversity for sustainable drug discovery that will benefit humanity and the planet.
